# Celebrating our journal’s commitment to strengthening primary health care research

**DOI:** 10.4102/safp.v67i1.6139

**Published:** 2025-04-16

**Authors:** Klaus B. von Pressentin, Arun Nair, Ramprakash Kaswa, Mareike Rabe, Mmaphefo Maluleka, Simon Marcus, Indiran Govender

**Affiliations:** 1 Department of Family, Community and Emergency Care, Faculty of Health Sciences, University of Cape Town, Cape Town, South Africa; 2 Department of Family Medicine, University of the Free State, Kimberley, South Africa; 3 Robert Mangaliso Sobukwe Hospital, Northern Cape Department of Health, Kimberley, South Africa; 4 Department of Family Medicine and Rural Health, Walter Sisulu University, Mthatha, South Africa; 5 Mthatha General Hospital, Mthatha, South Africa; 6 Vita Oncology Cape Gate and Bellville, Cape Town, South Africa; 7 Department of Family Medicine and Primary Health Care, School of Medicine, Sefako Makgatho Health Sciences University, Pretoria, South Africa; 8 Division of Family Medicine, University of the Witwatersrand, Johannesburg, South Africa

Research can strengthen the foundational knowledge of primary health care (PHC) as a universal framework and family medicine as a distinct clinical speciality. A recent report identified research gaps and priorities for African family medicine and PHC and called on journals, partners and stakeholders to strengthen primary care and PHC research in the African region.^1^ As the *South African Family Practice Journal* (SAFPJ) editorial team, we are committed to responding to this call. This editorial will describe some of our current capacity-building activities: a seminar on reporting guidelines and an introduction to our editorial fellowship.

## The importance of standardising research reporting

High-quality research that adheres to rigorous reporting standards is needed, and several reporting guidelines for health research are available via the EQUATOR (Enhancing the QUAlity and Transparency Of health Research) network.^2^ These standards enhance transparency, reproducibility and reliability, which benefit authors, reviewers and readers. During the September 2024 Congress of the South African Academy of Family Physicians (SAAFP) held in Cape Town, a seminar explored the role of reporting guidelines. Facilitated by the SAFPJ editors, the seminar focused on enhancing the quality of primary care research by examining the recently published Consensus Reporting Items for Studies in Primary Care (CRISP) checklist.^3^ The CRISP Working Group developed it over 5 years, engaging 300 participants from 29 countries.^3^ The EQUATOR network, the World Organization of Family Doctors (WONCA) and family medicine journals, including the *African Journal of Primary Health Care & Family Medicine* (PHCFM), endorse it.^4,5,6^

Since 2023, the editors and editorial board of the SAFPJ have interacted with the authors of CRISP several times and have agreed to recommend using the CRISP checklist as an optional step in 2024 for original research submissions to SAFPJ. The CRISP group has welcomed feedback on the checklist’s usefulness in Africa, and the SAFPJ editors saw this 2024 SAAFP congress seminar as an ideal opportunity to raise awareness of reporting guidelines and discuss the relevance of the CRISP checklist in our context.

### Unpacking different perspectives on reporting guidelines

Nineteen participants, from early-career researchers to seasoned academics, attended the seminar. *South African Family Practice Journal* editors started with an overview of reporting guidelines, highlighting their importance for authors, reviewers and readers. The EQUATOR network offers an online repository of checklists focused on study designs, health issues or disciplines (https://www.equator-network.org). The CRISP checklist was introduced as a framework tailored for primary care research reporting. Authors should adhere to reporting guidelines during article preparation, as employing checklists early enhances clarity, transparency and completeness, thereby improving the quality of the article and its chances of acceptance in reputable journals. Reviewers are vital in maintaining research integrity and should utilise reporting guidelines for structured feedback. Standardised reporting allows readers, including clinicians and policymakers, to appraise findings effectively.

### Should reporting guidelines be mandatory?

One of the seminar’s central debates was whether adherence to reporting guidelines should be optional or mandatory. While not yet a compulsory requirement for SAFPJ submissions, there was strong support for integrating the CRISP checklist into the journal’s instructions for authors. Participants agreed that mandatory adoption would elevate research quality, streamline the peer-review process and support emerging researchers in producing high-impact work.

### Capacity-building strategies

A recurring theme was the need for continuous education on reporting guidelines. Participants suggested integrating training into academic registrar programmes, hosting webinars and workshops as part of SAAFP’s ongoing training and creating links to the EQUATOR network on the journal’s site for access to global reporting standards. These measures would ensure that researchers, especially those new to scientific writing, are equipped with the necessary tools to meet international publishing standards.

### Strengthening research reporting – The way forward

The seminar concluded with a consensus on the significance of structured reporting in strengthening primary care research. The updated SAFPJ submission guidelines include the CRISP checklist as an optional but strongly recommended step. The SAFPJ editorial team is dedicated to improving research quality and transparency. We will continue supporting authors and reviewers by integrating structured reporting into primary care research practices.

## Growing the next generation of leaders and scholars

This engagement with reporting guidelines aligns with our commitment to advancing primary care scholarship in and for our SAAFP community. In 2025, we celebrate the 45th anniversary of the SAFP journal, established in 1980, the same year the SAAFP was founded. At the end of 2024, we launched a new editorial fellowship initiative to provide up to three South African family medicine registrars with the opportunity to collaborate closely with the journal’s editorial team for 1 year. Leading international family medicine journals have implemented similar initiatives.^7^ This fellowship is designed to help fellows achieve agreed-upon goals while gaining hands-on experience in scientific publishing. This fellowship initiative underscores our commitment to nurturing the next generation of family medicine and primary care leaders and scholars.
